# Supplementary material for: Are carbon emissions trading and green financial instruments synergistic? -Comprehensive quantitative research based on content analysis
Source: PLoS One. 2024 Mar 7;19(3):e0298601. doi: 10.1371/journal.pone.0298601 (PMC10919601; doi:10.1371/journal.pone.0298601)
Supplement: S1 Data — (ZIP) [file pone.0298601.s001.zip › Data/地级市Z_F工作报告.zip!/╔╜╢1⁄2/┴─│╟╩╨/└┤╫╘╣1⁄2╓┌║┼í╛┬φ┐╦╩2╛▌═°í┐_2006.docx]

各位代表：

现在，我代表市人民政府向大会作政府工作报告，请予审议，并请市政协委员和其他列席会议的同志提出意见。

一、“十五”期间政府工作回顾

“十五”时期是新世纪经济社会发展的重要时期。五年来，在省委、省政府和市委的坚强领导下，在历届领导班子打下的良好基础上，全市人民高举邓小平理论和“三个代表”重要思想伟大旗帜，深入贯彻党的十六大和十六届三中、四中、五中全会精神，全面落实科学发展观，认真贯彻省委、省政府的一系列重要部署，解放思想，干事创业，开拓进取，扎实工作，“十五”计划主要指标提前一年完成，全市改革开放和现代化建设取得新的成就，经济社会进入新的发展阶段。

过去的五年，是我市经济实现跨越发展、综合经济实力显著增强的五年。经济发展速度明显加快，经济结构不断优化，发展后劲日益增强，发展环境进一步改善，综合经济实力上了一个大台阶。2005年，全市生产总值达到689.1亿元，比2000年增加414.4亿元，五年翻了一翻多，年均增长15.4%；人均生产总值达到1480美元，年均增长14.8%。境内财政总收入和地方财政收入达到52.5亿元和26.2亿元，比2000年分别增加31.9亿元和13.8亿元，年均增长20.5%和32.4%。全市金融机构各项存贷款余额达到527.9亿元和415.9亿元，较2000年分别增加279.6亿元和215.3亿元。

过去的五年，是城乡面貌发生较大变化、人民生活水平明显提高的五年。创造性地提出并打响“江北水城”品牌，带动城乡面貌焕然一新。坚持以城带乡、城乡一体推进，城镇化水平由2000年的26.3%提高到2005年的34%。五年累计完成城建投资52亿元，其中聊城城区22亿元。城市基础设施明显改善，聊城城区及7个县市城区供水、供热、供气、污水处理、城市交通等功能逐步完善。五年累计城镇新增就业26.9万人，新增转移农村劳动力72.8万人。2005年，城镇居民人均可支配收入和农民人均纯收入分别达到9019元、3448元，比2000年增加3679元和1226元，年均增长11.1%和9.2%。城乡居民储蓄余额359.2亿元，年均增长12.8%。社会消费品零售总额233.6亿元，年均增长14.2%。城镇人均住宅建筑面积和农民人均生活用房面积分别达到30.8和33.2平方米。固定电话、移动电话、互联网用户分别达到98.2、156.5和6.8万户，年均增长11.7%、49.3%和31.5%。居民消费价格总水平保持在增长2%以内。

过去的五年，是改革开放取得重大进展、社会事业全面进步的五年。市场取向改革实现新的突破，社会主义市场经济体制逐步完善。全面推进对外开放，走出了具有自己特色的开放发展之路，参与国际竞争与合作的水平进一步提高。社会主义精神文明和民主法制建设稳步推进，教育、科技、文化、卫生、体育等社会事业全面发展，社会公共服务体系逐步建立，社会管理水平有新的提高。平安聊城、文明聊城、和谐聊城建设取得明显成效。全市政治安定，社会稳定，人民群众安居乐业。

五年来，我们主要做了以下工作：

（一）紧紧抓住第一要务，促进经济社会又快又好发展。面对复杂多变的形势，始终坚持以经济建设为中心不动摇，坚定不移地贯彻落实党中央、国务院和省委、省政府的决策部署，注重与聊城实际相结合，创造性地开展工作。根据市委提出的“跨越发展实现率先崛起，团结实干建设强市名城”的奋斗目标和“一二三四六”的工作思路，采取了一系列推动发展的重大举措。面对突如其来的非典和禽流感疫情，一手强化防控，一手抓经济不放松，取得了防控疫情和经济发展双胜利。牢牢把握宏观调控这一重大机遇，努力消除经济发展中的不稳定、不健康因素，着力调整经济结构、转变经济增长方式，着力深化改革、扩大开放，着力统筹城乡、区域和经济社会协调发展，确保了经济社会又快又好的发展。

（二）积极推进经济结构调整，提高经济运行质量和效益。不断调整优化第一产业，加快发展二、三产业，三次产业比例由2000年的33.5∶41∶25.5调整为18.6∶57.4∶24。把解决“三农”问题作为重中之重，全面落实各项支农惠农政策，较快地促进了农村发展和农民增收。2005年农业增加值达到128.1亿元，年均增长4.6%，林牧渔业增加值年均增长9.3%。粮食总产达80.2亿斤，创历史最高水平。在全国市一级率先开放发展科技特派员工程。加强农业基础设施建设，不断加大对农村和农业的投入，五年用于“三农”的财政性投入达15.1亿元。坚定不移地实施工业带动战略，全力打造百亿产业，积极推进“三个一批”，突出抓好“1030”工程和“5513”工程。2005年，全市规模以上工业实现销售收入989.3亿元、利税97.6亿元、利润62亿元，年均分别增长32.6%、31.5%和34.6%；实现利税过千万元的企业达到145家，其中过亿元的18家，比2000年分别增加101家和11家；创中国名牌5个、山东省名牌46个，驰名商标2个、著名商标42个。把发展服务业放到突出位置，大力发展旅游、商贸物流业，建设了香江大市场、新东方广场、人民广场、五星百货、金鼎商厦、新时代广场等大型商业设施，城市中心商业区基本形成，县（市、区）驻地的商贸设施水平明显提高。连续四年成功举办了“中国江北水城文化旅游节”和“水城之秋”观光周活动，2005年接待国内外游客441.3万人次，旅游总收入22亿元，年均分别增长17.1%和14.9%。

（三）全力抓好招商引资，努力扩大固定资产投入。把招商引资、增加有效投入作为经济发展的总抓手，大力实施“大开放、大招商，促进大发展”战略，招商引资取得重大成绩。五年来，招商引资到位资金累计达387亿元。韩国希杰、香港华润、日本洋马、四川希望等一批国内外大公司在我市投资、合作。五年累计完成固定资产投资1068亿元，年均增长32.8%，其中工业投入完成716亿元，年均增长44.4%，累计建成过亿元的项目154个，其中过10亿元的7个。新建改造国道、省道987.5公里，市驻地到各县市实现了高标准二级以上公路连接，基本建成了半小时经济生活圈。新增发电装机容量280万千瓦。实施了位山灌区改造，建设了彭楼引黄入鲁、谭庄水库、双海湖水库等一批水利设施。

（四）坚持不懈地培植“三个亮点”，着力解决经济发展的薄弱环节。积极把握我国加入世贸组织带来的机遇和挑战，创新招商引资方式，大力发展对外贸易。五年全市累计利用外商全部投资5亿美元，是“九五”的3倍，2005年利用外商直接投资1.1亿美元，是2000年的4.3倍，年均增长34.1%。2005年实现进出口总额6.4亿美元，其中出口4.6亿美元，是2000年的5倍，年均增长38.1%。成功举办了一系列境内外经贸活动，缔结了3个国际友好城市，对外合作交流不断扩大。加快发展高新技术产业，高新技术产业产值所占比重由2001年的10.7%提高到2005年的17.7%。大力发展民营经济，2005年民营经济增加值占生产总值的比重达到43.2%，比2000年提高了25.5个百分点。

（五）深化经济体制改革，不断增强经济发展活力。大力推进国有企业改革，多数国有大中型企业初步建立现代企业制度，国有中小企业改制基本完成。在全省率先推进国有资产管理体制改革。投资、价格体制改革取得新的进展。财税体制改革不断深化，市级实行了国库集中收付制度。加大对县乡财政转移支付力度，市财政五年累计转移支付5.2亿元，支持县乡发展，解决了多年积累的一些矛盾和问题。扎实推进农村税费改革，取消了农业特产税，停征乡村公益事业金等，大幅削减了农业税，减轻农民负担4.5亿元。金融体制改革不断深化，农村信用社改革进展顺利，城市信用社成功并入华夏银行。粮食、棉花流通体制改革有了新的进展。户籍制度改革稳步推进。国有土地市场化配置机制逐步完善，五年累计实现政府收益25.5亿元。城市公用事业改革有了重要突破。

（六）精心打造城市品牌，培植城市战略竞争力。坚持把中心城市作为区域发展的“龙头”，确立了建设卓越秀美“江北水城”的战略定位，按照“三高”的要求，加强城市规划建设管理，城市功能进一步完善，城市环境质量明显提升，“江北水城”已成为知名品牌，在国内外产生较大影响。完成了聊城城市总体规划新一轮修编工作。完成了东昌湖开发、水城广场、人民广场、运河开发、徒骇河城区段开发、东昌路改造、振兴路、机场路、光岳路、东昌大桥等一批城市精品工程，构筑起江北水城主体框架。积极开展“五城同创”，被评为中国优秀旅游城市和省级园林城市，创建国家卫生城和环保模范城取得重要阶段性成果。县市城区和小城镇建设取得重大进展，城镇面貌明显改观。五年新增城市绿地面积712.5公顷，新增城市道路2100万平方米，完成旧城改造拆迁面积560万平方米，新建住宅944.8万平方米，大大改善了城市居民的住房条件和生活质量。

（七）坚持以人为本，注重为群众办实事。加大扶贫力度，完成了16.2万农村贫困人口的脱贫任务。实施各种就业计划和行动，城镇登记失业率控制在4%以内。完善社保体系建设，不断提高城市低保人员待遇，切实做好“两个确保”。加强社会救助体系建设，发放救助资金1.8亿元，使10万困难群众得到救助。清理历年拖欠农民工工资10023万元。坚持不懈地抓了农村“路、水、电、医、学”建设，“村村通”工程投资16亿元，修建柏油路12105公里，通油路行政村比例达94%；投资1.7亿元用于人畜饮水解困和农村通自来水工程，有1139个村、108.3万农民用上安全卫生的自来水；投资15.2亿元实施农村电网改造；着重抓好了乡镇中心卫生院建设，积极推行新型农村合作医疗试点，有116.5万农民参合受益；投资4.7亿元改造农村中小学危房102万平方米，新建、改建农村中心中小学296处、70.6万平方米。

（八）坚持统筹协调发展，推动社会事业全面进步。认真实施可持续发展战略，积极推进资源节约和生态市建设，狠抓水污染和大气污染治理，实施了“四河一湖”碧水行动计划，关闭十五土（小）企业130家；积极控制农业面源污染，大力开展平原绿化和绿色通道建设，着重抓了城市绿化、美化，森林覆盖率达到25%，城市绿化率达到36.5%，比2000年分别提高9个和4.5个百分点。大力发展循环经济，4家企业被列为国家和省试点企业。认真实施科教兴聊战略，以开放的理念整合资源，走出了一条开放式发展科技、教育的路子。五年累计取得重要科技成果495项、授权专利1218件。大力推进与高校院所的合作，引进科技成果1096项，已成功实施612项。新增博士后科研流动站4家，省级以上技术研发中心5家，有6个项目进入国家“863”计划。全面落实教育优先发展的方针，“普九”水平进一步提高，创造出了茌平杜郎口“新课改”经验和冠县农村中小学后勤社会化改革、“民建公租”经验；职业教育、成人教育和民办教育进一步发展；聊城师范学院发展成为综合性大学，新成立了聊城大学东昌学院，聊城职业技术学院得到较快发展。全民健身活动蓬勃开展，体育健儿在全运会上实现了突破，成功举办了省第十一届大学生运动会和市第一届运动会，申办了第十一届全运会分赛场。高度重视人口和计划生育工作，年人口自然增长率控制在6‰以内。加强公共卫生“两个体系”和动物疫病防控体系建设，城乡预防、保健、医疗网络进一步健全。加快文化大市建设，聊城图书馆、水城明珠剧场、聊城杂技团、京剧院、豫剧团、中国运河文化博物馆等文化设施先后落成使用。国防动员、民兵预备役和双拥工作取得新成绩，被评为“全国双拥模范城”。不断加大对欠发达县、16个经济弱乡镇和莘县马西地区帮扶力度，推动区域经济协调发展。新闻出版、广播电视、社会科学、外事、侨务、对台、档案、妇女儿童、老年人、残疾人、民族、宗教、史志、地震、气象等工作都取得新的发展。

（九）积极开展平安聊城建设，大力优化发展环境。着眼于维护良好的社会秩序和公共安全，积极开展平安创建活动。深入开展严打整治斗争，加强社会治安综合治理，健全完善治安防范体系，治安案件明显下降，群众安全感增强。大力开展“四五”普法，推进依法治市进程。认真做好信访工作，妥善处理人民内部矛盾。积极化解了土地征用、城镇拆迁、企业改制、企业军转干部等方面的问题。高度重视安全生产工作，突出基层和基础建设，深入开展专项整治，安全生产形势平稳。应急管理体系和机制初步建立。加强市场秩序整顿和金融生态环境建设，诚信聊城建设取得明显成效。

（十）加强政府自身建设，提高政府行政水平。按照“建设好班子，造就好队伍，树立好政风，争做好公仆”的要求，以从严治政、狠抓落实为主线，加快阳光政府、责任政府、服务政府、法治政府建设。大力推进政府工作提速，深化行政审批制度改革，建立了市政府行政服务中心，实行了行政许可集中、联合、统一办理制度。大力推进政务公开，开通完善了政府网站，建立了新闻发言人制度，设立了市长信箱和市长公开电话受理中心，扩大了政府公报的发行范围。大力推进政府决策科学化、民主化，建立健全了决策目标、执行责任、考核监督“三个体系”，邀请人大代表、政协委员列席市政府会议，吸收知名专家参加重大决策咨询论证。稳步推进行政执法体制改革，积极开展相对集中行政处罚权工作。深入开展廉政教育和反腐败斗争，建立和完善各种制度，强化监察监督、审计监督和社会监督，从源头上预防和治理腐败，认真纠正部门和行业不正之风，查处了一批违法违纪案件。

各位代表！五年来，我市经济社会发展取得显著成就，我们在实践中探索积累了宝贵的经验。主要体会是：一定要坚持以科学发展观统领全局，始终抓住第一要务不放松，努力实现全面协调可持续发展；一定要坚持不断调整优化结构，转变经济增长方式，提高经济发展的质量和效益；一定要坚持深化改革，创新体制机制，坚定不移地走开放发展的路子，充分整合利用国内外资源，增强经济社会发展的活力和动力；一定要坚持以人为本，真心实意地为人民群众谋利益，让人民群众更多地享受改革发展成果；一定要坚持创造平安和谐诚信开放的发展环境，对外树立良好形象，对内增强凝聚力，充分调动干事创业的积极性；一定要坚持解放思想、与时俱进，求真务实、真抓实干，始终保持奋发有为的精神状态。

各位代表！五年来，中央和省领导同志多次视察聊城，特别是胡锦涛总书记2003年12月视察聊城，对聊城工作提出殷切希望，给全市人民以极大的鼓舞和鞭策。回顾五年历程，我们深深感到，聊城改革与发展取得的一切成绩，都得益于党中央的英明决策和亲切关怀，得益于省委、省政府和市委的正确领导，得益于各级人大、政协、社会各界的大力支持，得益于全市人民团结一心、艰苦奋斗。在此，我代表市人民政府向全市工人、农民、知识分子和干部，向关心和支持政府工作的各民主党派、工商联、无党派人士和社会团体，向驻聊人民解放军、武警官兵、公安干警和中央、省驻聊单位，向所有关心和支持我市发展的海内外朋友，向在我市创业的国内外投资者、合作者，表示衷心的感谢和崇高的敬意！

各位代表！在充分肯定这五年成绩的同时，我们也清醒地看到，我市经济社会发展还面临一些困难、矛盾和问题，主要是：经济总量小，结构性矛盾比较突出，经济增长方式较为粗放；有效投入仍然不足，发展的后劲还需大力培植；自主创新能力较差，科技进步比较薄弱；农业综合生产能力依然较弱，农民持续增收的长效机制尚未形成；服务业发展水平较低，消费需求拉动较弱；统筹城乡发展难度较大，社会事业发展任务艰巨；城镇化水平过低，区域之间、城乡之间发展不平衡；就业压力较大，社会保障能力不强；资源环境问题比较突出，对经济社会可持续发展构成较大影响；市场化程度不高，对外开放水平较低；各个层次人才短缺，人力资源尚未得到有效开发；社会建设和管理滞后，仍然存在一些不稳定、不安全因素；政府职能转变还不能很好地适应市场经济发展要求，依法行政能力有待进一步提高；一些地方和单位作风不扎实，工作效率低，公共服务意识淡薄；极少数人以权谋私，腐化堕落。对存在的问题，我们一定要高度重视，认真对待，采取有力措施，切实加以解决。

二、“十一五”时期经济社会发展的目标任务

从今年开始，将进入第十一个五年规划时期。根据党中央、国务院、省委、省政府和市委的统一部署要求，市政府在深入调查研究、广泛征求意见的基础上，编制了《聊城市国民经济和社会发展第十一个五年总体规划纲要（草案）》，业经中共聊城市委十届八次全会审议。《纲要（草案）》已印发大会，请一并予以审议。

“十一五”在我市全面建设小康社会、加快推进现代化建设的历史进程中，处于承前启后的重要阶段。这一时期，国际环境总体上有利于我们集中精力进行现代化建设，国内经济仍将保持平稳较快发展的态势，我市正处在人均生产总值跨越3000美元，工业化、城市化步伐加快的重要阶段，既面临难得的发展机遇，也面临严峻挑战。为此，抓住重要的历史机遇，规划实现跨越发展的宏伟蓝图，对于在新的历史起点上，实现新的发展，具有十分重大的战略意义。

“十一五”时期我市经济社会发展总的指导思想是：以邓小平理论和“三个代表”重要思想为指导，以科学发展观统领全局，全面贯彻落实党的十六大和十六届三中、四中、五中全会精神，深入贯彻落实省、市委确定的工作思路和发展目标，坚持又快又好发展这个主题，进一步增强自主创新能力和体制机制活力，大力推进结构优化升级、增长方式转变和统筹协调发展，加速推进工业化、城镇化和社会主义新农村建设，加快新兴工业城市、区域商贸中心城市和著名旅游城市建设，努力实现经济的持续快速协调健康发展和社会的全面进步，力争到2010年与全省总体上同步全面建成小康社会，2020年与全省总体上同步基本实现现代化。

综合考虑发展环境、发展阶段和基础条件，《纲要（草案）》从经济增长、统筹发展、资源发展、改革开放、人民生活等五个方面，提出了“十一五”期间的发展目标。即：在优化结构、提高效益和降低消耗的基础上，到2010年全市生产总值达到1400亿元，年均增长15%左右，人均生产总值3000美元。三次产业结构大体调整为10∶60∶30。固定资产投资年均增长20%，地方财政收入年均增长18%，社会消费品零售总额年均增长15%。“十一五”末单位生产总值能源消耗比“十五”末降低23%左右，高新技术产业产值占规模以上工业总产值比重达到25%。人口自然增长率年控制在7.5‰以内，城镇登记失业率年控制在4%以内。城镇居民人均可支配收入达到13250元，农民人均纯收入达到5070元，年均分别增长8%左右。

实现“十一五”规划的宏伟目标，必须从宏观上牢牢把握好以下几点：一是推动经济社会转入科学发展的轨道，务必迈出实质性步伐。这是经济工作总体要求的精神实质。要把它贯穿到经济社会发展的全过程，体现在方方面面。进一步转变发展观念，创新发展模式，促进经济社会又快又好发展。二是大力推进经济结构调整，加快转变经济增长方式。这是经济工作的主线。聊城经济质量、效益不高，财政比较困难，主要是结构性矛盾突出、增长方式粗放造成的。要下大气力抓好产业结构、投资结构、财税结构、金融结构、消费结构等一系列重大结构的调整，全面提高经济的整体素质。正确处理总量扩张、发展速度与提高效益的关系，更加重视发展的质量、结构和效益，从根本上转变经济增长方式，走出一条科学发展的道路。三是着力提高自主创新能力，增强区域经济竞争力。这是经济工作的一项重大战略任务。要大力提高原始创新能力、集成创新能力和引进消化吸收再创新能力，在重点领域实现突破，构筑区域性科技创新体系，再造区域经济发展新优势。四是深入推进改革开放，增强经济社会发展活力。这是经济社会发展的重要举措。当前改革仍然处在攻坚阶段，必须加大改革力度，不断突破制约经济社会发展的体制、机制性障碍。开放是聊城的必然选择，必须加大开放力度，在招商引资、三次产业、社会事业等方方面面，都要走开走好开放之路。五是高度重视资源环境问题，加快建设资源节约型和环境友好型社会。这是对历史负责、对子孙后代负责的重大责任和义务。要把节约资源、保护环境放在重要战略位置，着力提高资源利用效率、降低物质消耗、保护生态环境，创造良好的人居环境，真正实现经济与资源、环境的协调可持续发展。六是更加重视社会事业发展，推进和谐社会建设。这是经济社会发展的根本目的。要坚持以人为本，从关系人民群众切身利益的现实问题入手，增加公共财政支出，突出解决好就业、社保、教育、卫生、环保、安全等问题，切实让人民群众享受到改革发展的成果。统筹协调各方面的利益关系，妥善解决各种矛盾，加快建设平安聊城、文明聊城、和谐聊城，促进经济、政治、文化、社会四位一体协调发展。

各位代表！经过五年的艰苦努力，我市经济社会一定会在转入科学发展的轨道上迈出新的步伐，全市的综合经济实力一定会进一步增强，生态环境一定会有明显改善，人民的生活水平和质量一定会有较大提高，一定会与全省总体上同步建成全面小康社会。在此基础上，再经过十年的努力，我们就能与全省总体上同步基本实现现代化。这个宏伟目标和美好前景，将召唤全市人民在社会主义现代化的道路上奋勇前进！

三、2006年全市经济社会发展的重点

2006年是实施“十一五”规划的开局之年，做好今年的工作意义十分重大。我们要按照中央和省委、省政府的决策部署，按照“十一五”规划的目标任务，以科学发展观统领全局，认真落实中央宏观调控政策，继续实施市委确定的“一二三四六”的发展目标和工作思路，推动经济社会切实转入科学发展的轨道并迈出实质性步伐，努力实现又快又好的发展，确保“十一五”开好局、起好步。综合考虑各方面因素，经济社会发展的主要预期目标是：全市生产总值增长15%；万元生产总值能耗降低6.5%；社会商品零售额增长15.5%；高新技术产业产值占规模以上工业总产值的比重达到19.3%；全社会固定资产投资增长30%，达到460亿元；外商直接投资增长20%，达到1.3亿美元；外贸出口增长20%，达到5.5亿美元；地方财政收入增长18%，突破30亿元；城镇登记失业率控制在3.6%以内；城镇居民人均可支配收入增长10%，农民人均纯收入增长8%；人口自然增长率控制在7.5‰以内。

今年要着重抓好以下十个方面工作：

（一）高度重视“三农”工作，扎实推进社会主义新农村建设

坚持把解决“三农”问题作为重中之重，认真贯彻“城乡统筹发展、工业反哺农业、城市支持农村”的方针，按照“生产发展、生活宽裕、乡风文明、村容整洁、管理民主”的目标，积极推进社会主义新农村建设，努力实现良好开局。

加快发展农村经济。继续实行最严格的耕地保护制度，稳定粮食总产，抓好优质粮食基地建设。加大农业结构调整力度，大力发展畜牧、蔬菜、水果、水产、林木、花卉等高效农业，加快发展养殖大户和规模饲养场。新增无公害农产品基地20万亩、绿色食品基地6万亩。大力推进农业机械化、标准化、产业化、国际化。市、县两级建立农产品质量监测中心。抓好高致病性禽流感等重大动物疫病的防控工作。积极发展农村二、三产业，推进产业化经营，力争新增1家国家级、5家省级以上龙头企业。切实抓好农产品流通，在聊城城郊高标准规划建设一处大型农产品批发交易中心，使我市更多的农副产品打入京津济沪等大城市的批发市场和超市。

深入推进科技特派员工程。坚持开放理念，积极引入国内外科技资源，深入推进科技特派员工程。今年全市科技特派员要增加300人以上。充分发挥专修学院和法人科技特派员的作用，搞好对科技特派员的培训。扎实搞好20个重点示范基地建设，每个乡镇建立2-3个不同领域的示范基地。着力扩大科技特派员工程领域，促进农村二三产业发展。

加强农村基础设施和村镇规划建设。加大公共财政对农业和农村发展的投入，今年市级财政安排支农资金2.25亿元，增长12.4%。鼓励和引导各种社会资本投向农业和农村。新建改造农村公路572公里，新增50万农民用上自来水，新增节水灌溉10万亩，改造农村危桥100座，建设完善农村供电、电信等基础设施，农村客车通达率达到99%。发展生态家园沼气工程2000个、户用沼气工程5000户，在有条件的村积极推广太阳能。高水平进行村镇规划、建设和管理，推广节材、节能、美观、舒适的新型农村住宅，每个县（市、区）抓好3－5个试点村。

全面深化农村改革。继续深化农村税费改革，全面取消农业税，推进乡镇机构、农村义务教育等改革，切实减轻农民负担。建立财政支农资金稳定增长机制。在稳定完善农村土地承包关系的前提下，引导和规范农村土地承包经营权流转。加快农村小型基础设施产权制度改革。积极化解乡村债务，杜绝发生新的乡村债务。鼓励农民在自愿的基础上，组建新型农村合作经济组织。完善村民自治机制，发展农村基层民主政治，保障广大农民的民主权利。

大力培养社会主义新型农民。充分利用各种教育资源，抓好“阳光培训”、绿色证书培训和新型农民科技创业培训。积极开展和谐家庭、和谐村组、和谐村镇创建活动，倡导科学健康、文明向上的生活方式。加快县级文化馆、图书馆和乡镇文化站、村文化室等农村公共文化设施建设，鼓励农民兴办文化产业。深入开展“三下乡”活动，丰富农村精神文化生活。多种形式对农民进行党的政策和移风易俗宣传教育，普及科学、法律知识，搞好乡规民约建设。

建设社会主义新农村，是一项长期的历史任务。各级政府必须从实际出发，坚持规划先行，抓好试点示范，有步骤、有计划、有重点地逐步推进。实际工作中要充分尊重农民意愿，严禁强迫农民去干不愿意干的事情，让农民真正受益，切实把好事办好！

（二）加快经济结构调整，努力转变经济增长方式

紧紧抓住结构调整这条主线，大力调整二、三产业结构，积极发展非公有制经济，加快转变经济增长方式，增强全市经济的整体素质和综合竞争力。

加快工业经济结构优化升级。坚定不移地实施工业带动战略，按照“三个一批”的要求，以“5513”工程为抓手，全力建设百亿产业，发展产业集群，努力走出一条有聊城特色的新型工业化道路。坚持大上项目、上大项目，围绕核心企业，着力发展具有比较优势的电力及有色金属、汽车及农业装备、林浆纸业、医药、食品、化工及化肥、纺织服装等七个产业链，吸引战略投资者合资合作，延伸产业链条，打造产业集群，带动中小企业发展。以信息化带动工业化，加大用高新技术改造传统产业力度，骨干企业新上项目都要努力走高、精、尖的路子。突出抓好涉农骨干企业的调整优化，在巩固现有基础和市场份额的同时，大力发展高新技术和高附加值产品。加强对国内外大企业典型个案研究，突出加强企业核心竞争力建设，夯实技术、质量、管理、品牌基石，争做百年企业。加强对煤、电、油、运的调度，保证工业经济平稳运行。改善企业融资担保结构，防范化解关联风险。加强企业家队伍建设，鼓励引导企业家不断进取、不断超越。力争今年“5513”工程企业销售收入有2户过100亿元、2户过50亿元，利税过千万元的企业达到180户、过亿元的20户。力争新创省级名牌10-15个、国家名牌2-3个，使通过ISO9000质量体系认证的企业新增30家以上。

着力繁荣发展服务业。把服务业作为优化结构、推进城市化、安置就业、增加财政收入的重大战略重点来抓，在改革服务业体制、改善政策环境、社会环境、法制环境和培植服务业集团、打造名牌方面取得实质性进展。大力发展旅游业，继续突出江北水城和历史文化名城特色，深刻挖掘文化内涵，尽快把聊城培育成全国旅游热点城市。加快推进古城恢复性开发等重大旅游项目建设，形成有吸引力、震撼力的大景点、大景区，努力争创3A级、4A级景区景点。尽快推出“江北水城精华游”，融入我省“山、水、圣人”精品旅游线。继续打造“夜游水城”品牌，建设特色旅游街区。继续办好江北水城文化旅游节和水城之秋观光周活动。大力发展现代流通业，把聊城建设成具有较强辐射能力的区域性商贸物流中心。制定全市流通业发展规划，形成布局合理的流通网络。着力抓好市中心商业区、城区北部物流园区、城区东部物流带和城区南部商业区的发展。加快在城区北部建设大型物流基地，重点搞好煤炭、矿石、农副产品等物资、产品的存储运销。引进先进经营理念，培植3-5个大型骨干商贸流通企业。积极发展金融保险业，提高地方金融机构竞争能力和服务水平，吸引辖外金融保险和投资机构来聊设立分支机构。规范发展房地产业，激活住房二级市场。大力发展交通运输、信息服务、社区服务、中介服务等行业，全面提升第三产业发展水平。

大力发展非公有制经济。认真落实国务院鼓励支持和引导个体私营等非公有制经济发展的若干意见，着力营造平等竞争的法制环境、政策环境和市场环境。加快民营企业实现投资主体和产权多元化，鼓励进入公共事业、基础设施等领域。实施中小企业成长计划，筛选100家企业重点加以扶持。大力抓好融资担保、信用评价、技术服务、人才培训、法律服务、行业协会等服务体系建设，尤其在拓宽中小企业融资担保渠道上实现大的突破。

推进区域经济协调发展。认真研究融入济南都市圈的战略规划，加快与济南接轨的步伐。抓住国家大力发展天津滨海新区和北京承办2008年奥运会的机遇，主动承接环渤海发达地区的辐射和产业转移。大力发展以临清、高唐、茌平组成的北部综合经济区，以东昌府、开发区和东阿组成的中部综合经济区和以阳谷、莘县、冠县组成的南部综合经济区。进一步扩大县级经济管理权限，加快县域经济发展。经过3-5年努力，高唐、临清、茌平力争进入全省30强；东昌府、阳谷实现位次前移；东阿、莘县、冠县尽快摆脱弱县局面。大力抓好15强乡镇和16弱乡镇的发展。继续加大对莘县马西地区、黄河故道、沉沙地区、金堤河滞洪区等区域的帮扶力度，使其尽快改变贫困落后面貌。

（三）切实增加有效投入，下大气力抓好招商引资

在今后相当长的一个时期，保持较高的投入增长，是我市加快工业化、城市化的必由之路，是不可逾越的发展阶段。要把抓有效投入、培植新的经济增长点，作为经济工作的首要任务，力争在今年有较大突破。

全力抓好招商引资。把招商引资作为经济工作的总抓手，紧紧围绕重点产业、核心企业抓招商，围绕延长产业链条、增加财政收入抓招商。充分发挥企业主体作用，注重加强专业招商队伍建设。进一步改善投资环境，建立完善的项目落地机制，提高招商成效。继续把日韩、港台、欧美和长三角、珠三角作为重点招商地区。集中力量引进投资过十亿元的大项目、世界500强项目和高新技术项目。全市招商引资1000万元以上项目到位资金力争实现60亿元。把开发区和园区作为招商引资的重要载体，举全市之力发展聊城经济开发区，各县（市、区）园区在产业发展上要有所侧重、各具特色，增强竞争力。

大力拓宽投融资渠道。积极搭建银企合作平台，继续办好银企合作推介会，落实好签约项目资金。鼓励商业银行进一步加大信贷支持力度，对贷款需求量大的项目，采取银团贷款方式。充分发挥企业上市、股份改造、产权出让、发行债券、票据融资、产业发展基金和创业投资基金等渠道的作用，争取在企业上市融资方面实现突破。

抓好重大项目投资。积极做好重大项目的筛选、储备和争取工作。重点抓好凤祥集团阴极铜、信发集团铝深加工、国电二期2×60万千瓦机组、奥博特高精度铜合金板带、希杰集团生物工程、泉林集团林浆纸一体化、时风集团轻卡汽车、冠洲集团“三板”、鲁西化工工业园、莘县盐化工等重大项目的建设。新建改造干线公路203公里，积极争取德商高速公路、邯济铁路复线等项目尽早开工。抓好位山灌区续建配套工程和沉沙池区、金堤河、马颊河的治理，配合国家搞好南水北调东线工程建设。切实加强对项目建设的督导和管理，确保重大项目早日建成并发挥效益。

努力优化投资结构。大力发展符合国家产业政策、节约能源资源的项目，发展高新技术和先进制造业项目，发展一批重大基础设施和社会事业项目。严格禁止新上高耗能、高耗水、高污染项目，严格限制新上产能过剩、低水平重复建设特别是在市域范围内恶性竞争的项目。

（四）切实提高自主创新能力，建设创新型城市

全面落实全国科学技术大会精神，把加快科技进步、提高自主创新能力，作为增强经济核心竞争力的中心环节和关键所在。坚持开放发展科技，整合吸引国内外科技、人才资源为我所用，致力于形成良好的创新文化和创新环境，努力走出一条符合聊城实际的自主创新之路。

加快推进科技自主创新。一是大力发展高新技术产业。以电子信息、新材料和生物工程为重点，培植高新技术产业群。加快启动高新技术产业园建设，使之尽快成为我市高新技术产业的重要载体。集中力量实施电动客车等一批重大科技专项。确保今年高新技术产业产值占规模以上工业总产值的比重比去年提高1.6个百分点。二是打造好科技发展平台。吸引国家著名科研院所和高校在聊城建设联合研究院，加快建设市院士工作站。努力创建国家级高新技术创业服务中心。充分发挥聊城大学等驻聊高校科研单位的作用，继续开展同国内重点高校院所的合作。三是着力培育自主创新的主体。建立以企业为主体、产学研结合的技术创新体系，鼓励支持企业与高校院所联合建立研发机构和博士后流动站。鼓励企业购买大院大所，大力开发具有自主知识产权的关键技术和核心技术。重点培育10户具有自主知识产权的企业技术中心。四是加大对科技的投入。今年市县两级财政要增加技术研究与开发资金投入，达到同级财政预算支出的1%以上。设立市高新技术创新示范工程专项资金、市科技型中小企业技术创新基金、市科技交流与合作专项经费，围绕最有希望的高科技项目集中投入。支持企业加大科技投入，今年骨干企业技术开发经费占销售收入的比重不能低于3%。

大力实施人才强市战略。抓住培养、引进、使用三个关键环节，加强人才资源能力建设。加快实施“2621”人才工程。加强优秀干部队伍和企业家队伍的培养，组织推荐到发达城市和知名院校学习、培训。整合职业教育资源，加大高级职业技术人才的培养力度，实施“千名技师培训计划”。全面落实吸引高层次人才的暂行办法，面向全国招聘优秀企业经营者、管理者和技术人才。吸引聊城籍研究生、大学生回乡发展，确保回聊工作的比去年增长15%以上。

积极推进国民经济和社会信息化。加强通信网络基础设施建设，推进通信网、计算机网络和广播电视网三网融合。加快村村通有线电视进程，推进数字化建设。积极开发利用信息资源和信息技术，发展软件业与信息服务业，加快利用信息技术改造提升传统工业、农业和服务业，推进政务、商务和社会公共服务信息化。

（五）进一步推进改革开放，增强经济社会发展活力

把改革放在更加突出的位置。紧紧抓住影响经济社会发展的突出矛盾和问题，创新体制机制，力争在一些关键领域和重要环节取得实质性突破。一是深化国有企业和国有资产管理体制改革。出台《聊城市国有经济布局调整指导意见》，促进国有资本在流动重组中提高质量、增加效益。大力引进战略投资者，实现投资主体多元化。积极推进东胶股份公司、中通客车公司的股权分置改革。规范完善中小企业改革改制工作。切实做好破产企业的重组、生产启动和职工安置。健全完善国有资产监督管理体制，切实搞好国有资产收益的收缴和管理，确保国有资产的安全和保值、增值。全面做好国有粮食购销企业的改革，进一步深化公用事业改革。二是深化财税价格体制改革。继续深化部门预算、国库集中收付、政府采购、收支两条线和县乡财政体制改革。依法规范税收和非税收入征管。加快构建公共财政体系，加大公共产品、公共服务设施和公益事业投入。完善水、气等资源性产品的价格形成机制，深化药品和医疗服务价格改革，规范教育收费行为。三是深化投资体制改革。切实突出企业投资主体地位。认真落实市里出台的投资管理暂行办法、投资项目审计监督办法，健全政府投资决策责任制度，完善重大项目专家咨询制度和后评估制度。加强部门之间的协调配合，形成建设项目核准与产业准入、土地供应、环保评价相互配套的联动机制。四是深化金融体制改革。稳妥推进农村信用社改革，完善法人治理结构。推进金融生态环境建设，建立地方金融风险预警机制，切实防范化解地方金融风险。五是深化事业单位改革。加快建立政事职责分开、法人地位明确、单位自主运营、政府依法管理的新型管理体制。对从事生产经营和开发服务的事业单位改企转制，全面推行事业单位人员聘用制。在抓好上述几项重大改革的同时，全面推进各部门、各单位、各企业自身的改革，靠改革增活力、增效率、增效益。

把外经贸作为第一亮点来抓。充分利用两个市场、两种资源，全面提高对外开放水平。一是积极扩大利用外资规模，提高利用外资质量。抓好落地一批、签约一批、在谈一批、新谈一批的落实。引导外资更多地投向高新技术产业、先进制造业、基础设施、现代服务业、现代农业和环保产业等，鼓励外资参与国有企业改组改造。推进更多的民营中小企业利用外资。争取引进几个利用外资过5000万美元的大项目。二是加快转变对外贸易增长方式。大力调整出口产品结构，巩固禽肉、纺织品和机电产品出口，扩大具有自主知识产权的产品出口。着力抓好出口大户特别是“5513”工程企业出口。组织企业积极参加国内外展洽活动，巩固日韩、港台市场，扩大欧美市场，大力开拓非洲、拉美市场。积极开展加工贸易，提高加工深度，延长增值链条。扩大紧缺资源和先进技术、关键设备进口。建立完善反倾销应诉支持机制和出口产品预警机制，提高应对国际摩擦的能力。加快建设聊城市保税物流中心和陆路口岸，建立符合国际惯例的配套物流服务体系，对进出口货物实行属地报关报验。三是大力实施“走出去”战略。在更大范围、更高层次上参与国际分工合作，避开国际贸易壁垒，带动资本、产品、劳务输出。实施资源来源多元化战略，鼓励茌平信发集团、阳谷祥光铜业等企业，在国外建立稳固可靠的铝矾土、铜精矿等资源基地。积极探索以“工程换资源”等新型对外承包工程形式。要推进各领域的对外开放，把全市的对外开放工作提升到一个新水平。

（六）积极扩大消费需求，进一步增强对经济社会发展的拉动力

把扩大消费需求作为经济社会发展的基本立足点和新的经济增长点，努力增加城乡居民收入，拓展消费领域，改善消费环境，促进消费结构优化升级。一是千方百计增加城乡居民收入。健全与经济发展水平相适应的收入增长机制，努力增加城乡居民收入，进一步提高购买力。落实国家出台的各项增资政策，进一步提高城乡低保水平，督促各类企业按照效益增长定期增加职工工资。规范住房、教育、医疗、保险等改革，切实改善居民消费预期，增强消费信心，激活即期消费。二是充分发挥中心城市、次中心城市的消费拉动作用。调整商业结构和经营模式，积极培育住房、汽车等高端消费，大力发展旅游、文化、娱乐、健身等热点消费。更多地吸引济南等大城市居民来聊城休闲消费，更多地吸引农民进城消费。三是努力扩大农村消费。实施“万村千乡”市场工程，以茌平、临清、莘县三个县（市）为试点，推动中邮物流等企业开展超市、连锁店下乡进村，力争用三年的时间使标准化的“农家店”覆盖全市80%以上的乡镇和60%以上的村庄，促进城乡消费结构有机接续。四是调整优化消费环境。研究出台鼓励消费的政策规定，创造支持消费的法制环境、诚信环境、政策环境、服务环境。积极发展信用消费。大力整顿和规范市场秩序，完善价格和收费公示制度，坚决打击制假售假、商业欺诈、偷逃骗税等行为，切实保护消费者的合法权益。

（七）加快城镇化进程，进一步提高城市规划建设管理水平

进一步拉开城市发展格局，逐步建立起以聊城市区为核心、各县市城区为次中心、辐射带动小城镇发展的组团式城市群。在城镇建设上，坚持高起点规划、高标准建设、高效能管理，着力在精细上下功夫，在优化环境、提高品位上下功夫，带动城市规划、建设和管理全面上层次、上水平。一是重点抓好中心城市建设。按照“一城五区”的总体规划和东扩南展的战略布局，搞好详规编制，加快构筑江北水城主体框架，提升江北水城的品位。适时启动南部行政区建设，积极稳妥地搞好古城区保护和开发，完成运河四期工程，实施徒骇河城区段开发，实现运河与徒骇河的贯通。实施东南湖沿岸整治、青年渠治理、新水河改造工程，进一步完善城市水系格局。实施建设路、花园路、利民路、陈口路建设改造。抓好城市供水、供热、供气等设施建设。二是高度重视县城和中心镇建设。科学规划，突出特色，加强基础设施建设，完善城市功能，搞好环境综合整治，继续构建完善半小时经济生活圈，逐步实现与中心城市一体化发展。三是努力提高城镇建设管理水平。大力实施“精细”工程。小到一草一木一石，大到楼房、街道、广场，都做到精、深、细、实，加快城市建设由低层次、低水平、粗放式向高品位、高质量、精细化转变。依法加强城市管理，全面提高城市执法水平。四是集中力量改善城镇环境。抓好国家卫生城、环保模范城和国家园林城的创建工作。搞好绿化、净化、美化和亮化，建设城市森林和城市公园，启动旧州洼湿地公园建设。彻底解决城市扬尘过多、违章建设、乱停车辆、乱摆摊点、占道经营等问题。继续搞好城中村改造、背街小巷的硬化和城郊结合部的整治。城市化水平提高到37%。

（八）构建资源节约型和环境友好型社会，加快生态市建设

大力发展循环经济。坚持节能降耗，节约资源，加快形成节约型增长方式。综合运用土地、环保、价格、信贷和税收等手段，严格落实《聊城市资源节约标准》。大力抓好机械、电解铝、电力、化工、轻工、建材、纺织等行业的技术改造和节能降耗，抓好粉煤灰、金属冶炼废渣、引黄沉沙、污水、农副产品加工废料的控制管理和综合循环利用。推行城市分类供水、中水回用和一水多用，建立用水累进加价制度，推广节水型农业。提高土地集约利用水平，开发区每亩投资要在150万元以上，各县（市、区）工业园区每亩投资要在100万元以上。积极推行清洁生产，在重点企业、工业园区和城市开展循环经济发展试点，鼓励产业循环式组合、企业循环式生产。从今年开始，市里将定期公布各县（市、区）单位生产总值、单位工业增加值能耗及污染物排放情况。

积极推进生态市建设。坚持预防为主、综合治理，全面推行排污许可证制度，从源头上防治污染和保护生态。实施“聊城数字环保”工程，定期公布环境质量、环境管理和污染治理信息。积极推进生态市、生态县、生态示范区建设，开展环保模范城市、环境优美乡镇、生态村以及生态社区、绿色学校等多层次的创建活动。以“四河一湖”碧水行动为重点，推动重点流域污染防治。严格环评标准，对环保不达标的生产线坚决关停，确保重点流域和区域环境质量明显改善。加快实施农业“两减三保”计划，抓好农业面源污染治理。强化大气污染治理，加快各电厂脱硫工程建设和工业粉尘治理力度，推广使用车用乙醇汽油。加快聊城垃圾处理厂和茌平、冠县、莘县、阳谷、聊城二期、临清二期污水处理厂工程建设。以城区、滩区、沿路、沿河和环城、围村为重点，大力开展植树造林，绿化覆盖率达到26%。

（九）统筹经济社会发展，加快发展各项社会事业

按照科学发展观的要求，统筹发展各项社会事业，不断改善人民群众的学习、生活条件，努力促进人的全面发展，提高全社会的文明素质。

优先发展教育事业。全面贯彻落实《义务教育法》，继续完善“以县为主”的农村义务教育管理体制。推广杜郎口经验，全面深化教育教学改革，培养学生的创新精神和实践能力。巩固提高“普九”水平，把辍学率严格控制在2%以内。从今年起，用两年的时间对全市农村义务教育阶段学生免收学杂费，对贫困家庭学生提供免费课本和寄宿生活费补助。完成城区14所小学新建扩建任务，抓好55所农村中心中小学建设。积极倡导民建公租和后勤社会化改革，鼓励民间资金投资教育事业。落实好公用经费，确保教师工资不低于当地公务员工资，并按时足额发放。扩大高中阶段教育规模。实施中等职业教育发展专项建设计划，每个县（市、区）建设改造一所高水平职业技术教育中心。大力支持高等教育发展，为聊城大学、聊城职业技术学院创造良好的办学环境。积极发展成人教育、继续教育和老年教育，加快建设学习型社会。

高度重视卫生事业发展。全面完成“两个体系”18个项目建设。切实做好艾滋病、乙型肝炎等重大传染病预防控制工作。推进城市医疗服务体系改革，加快发展社区卫生服务。扩大新型农村合作医疗试点范围，参合人数达到200万人。全面完成30所乡镇中心卫生院建设改造，加强乡镇普通卫生院建设，启动600个农村卫生室建设。合理配置医疗卫生资源，加强对困难群众的医疗救助，逐步解决群众看病难、看病贵的问题。积极推进中医药事业发展。

扎实做好人口与计划生育工作。稳定计划生育政策和低生育水平，积极平抑第四次人口高峰。加快建立计生工作新机制，强化基层基础工作，改善人口结构，提高人口质量。

积极发展文化、体育等各项社会事业。积极稳妥地深化文化体制改革，全面加强文化大市建设。引导、鼓励投资文化产业，打造演出市场品牌。积极发展城市社区文化和乡村文化。加强历史文化名城和文物保护，争取将聊城纳入全省特色文化名城建设计划，把文化名城——聊城推向世界。鼓励发展竞技体育，积极备战第二十一届省运会。加强城乡社区体育设施建设，引导开展全民健身和群众性体育活动。加快筹建市体育中心及第十一届全运会分赛场场馆，积极申请国家在聊设立水上运动训练基地，申请承办具有水城特色的国际赛事。大力开发体育产品，发展体育经济。切实搞好国防动员、人民防空、交通战备和民兵预备役建设，做好军队转业干部、退役士兵安置工作，积极支持驻聊部队建设，扎实开展双拥共建活动。进一步做好妇女儿童、残疾人、老龄以及慈善事业。大力做好广播电视、新闻出版、社科研究、民族、宗教、外事、侨务、对台、档案、史志、气象、测绘、防震减灾等各项工作。

（十）切实关心群众生活，努力建设和谐社会

牢固树立以人为本的思想，倾注真情为人民群众办实事、解难事，公平保障人民群众的政治、经济和社会利益，努力构建民主法治、公平正义、诚信友爱、充满活力、安定有序、人与自然和谐相处的社会主义和谐聊城。

加强社会主义精神文明和民主法制建设。大力开展爱国主义教育，深入开展群众性精神文明创建活动。积极推动“诚信聊城”建设。完善基层民主管理制度，进一步推行政务公开、厂务公开、村务公开。认真办好人大代表议案、建议和政协委员提案。深入开展普法宣传教育，大力推进社会管理法制化和行政执法规范化。做好法律服务和法律援助工作。

千方百计扩大就业。全面落实各项就业扶持政策。引进和发展劳动密集型企业，提高安置就业能力。探索建立城乡一体化的人力资源市场，加强就业培训和创业培训。大力开发公益性岗位，重点解决“4050”下岗人员、失地农民的再就业问题。今年力争新增城镇就业7万人。继续大力发展劳务经济，转变劳务输出理念，稳定输出总量，提高劳务素质，打造聊城劳务品牌，发展特色劳务，获取更多劳务收入。以建立鲁西海员基地和向日本、韩国派遣研修生为突破口，积极开拓国际劳务市场。今年市外就业人员稳定在100万人左右。积极鼓励外出务工人员回乡创业。

加快完善社会保障体系。扩大养老保险覆盖范围，逐步做实养老保险个人帐户。加强社保基金的收缴和管理运营，确保基金保值增值，确保各项社会保险金按时足额发放。严格执行城市低保标准和企业职工最低工资标准。稳妥推进企业离退休人员社会化管理。认真落实优抚政策，大力发展慈善事业。加快农村社会保障制度建设，逐步建立农村居民最低生活保障制度和失地农民基本生活保障制度。做好受灾群众救济工作，切实保障基本生活。

大力改善群众生产生活条件。更加重视关心社会弱势群体，建立健全城乡低收入困难群众的社会救助体系。逐步建立以财政为主的农村五保户供养机制，今年新建农村中心敬老院10处，改扩建11处，使五保集中供养率达到41％。继续实施“安康居住”工程，新建10万平方米经济适用房和廉租房。加快推进城镇居民供暖、供气工程建设，城区燃气发展居民用户6500户，新增供热面积250万平方米。建立长效机制，防止农民工工资发生新的拖欠。

扎实推进平安聊城建设。加强社会治安综合治理和治安防控体系建设，积极预防和妥善处置群体性事件，依法严厉打击各种刑事犯罪。正确处理人民内部矛盾，认真做好人民群众来信来访。高度重视处理好征地拆迁、企业改制等涉及群众切身利益的问题，坚决纠正各种侵害群众利益的行为。强化安全生产基层基础工作，狠抓道路交通、危险化学品、烟花爆竹、人员密集场所、电力、建筑施工和燃气管道等各项安全工作，形成安全生产长效机制，严防各类重大恶性事故的发生。尽快制定市、县、乡三级突发公共事件应急预案，提高保障公共安全和处置突发事件的能力。

四、切实搞好政府自身建设

面对新的形势和任务，各级政府必须按照科学发展观和建设和谐社会的要求，全面加强自身建设，不断提高行政能力和管理水平。

（一）加快转变政府职能，努力建设法治政府。认真贯彻依法治国基本方略，全面实施国务院颁布的依法行政纲要，努力做到依法行政、程序正当、高效便民、诚实守信、权责统一。坚持向人大及其常委会报告工作和向人民政协通报情况制度，自觉接受人大及其常委会的监督，政协、各民主党派和人民团体的监督，广大人民群众和新闻舆论监督。进一步加强和改善行政执法工作，做到严格执法、文明执法、公正执法、高效执法。继续深入开展相对集中行政处罚权工作。强化行政执法监督，健全行政执法人员责任追究制度。进一步深化行政管理体制改革，切实把政府职能转到经济调节、市场监管、社会管理和公共服务上来。改进政府管理经济的方式方法，努力提高按市场经济规律管理经济的能力，提高按国际通行规则办事的能力，提高运用经济法律手段调节经济的能力。强化服务意识和服务职能，继续推进政府工作提速，努力提高办事效率和服务水平。

（二）健全完善“三个体系”，着力提高行政效能。大力推进决策科学化、民主化，健全决策目标体系。改进决策规则和程序，完善重大决策调研制度、专家咨询论证制度和社会公示听证制度。对超越权限、违反法定程序造成重大损失的决策行为，严肃追究决策者的责任。大力推进执行责任的具体化、明确化，健全执行责任体系。完善目标责任制和主办负责制，做到工作主体清晰、责任明确、权责一致。大力推进考核监督的制度化、规范化，健全考核监督体系，对不作为、影响行政效能、贻误行政工作等行为，严肃追究部门负责人和当事人的行政责任。

（三）大力推进政务公开，切实转变工作作风。要从人民群众普遍关心的问题入手，全面推行公开办事制度。所有行政审批事项都要纳入行政服务中心，努力打造一流的行政服务窗口。切实办好市长公开电话，完善政府新闻发言人制度。加快电子政务建设，完善政府门户网站。深入贯彻落实《公务员法》，下大气力提升政府公务员的整体素质和水平。牢固树立科学发展观和正确政绩观，大力发扬求真务实的优良作风，说实话、办实事、求实效，力戒形式主义，力戒做表面文章。打造学习型队伍，构建多层次学习网络。切实精简会议和文件，更多地深入基层、深入群众调查研究，增强工作的针对性和实效性，把各项工作提高到一个新水平。

（四）始终坚持从严治政，狠抓队伍廉政建设。坚持标本兼治、综合治理、惩防并举、注重预防的方针，建立健全教育、制度、监督并重的惩治和预防腐败体系，深入开展反腐败斗争。认真落实党风廉政建设责任制，严格履行廉政承诺。教育政府公务员严于律己，克己奉公，清白清廉做人，公平公正做事。充分发挥监察、审计等监督机关的作用，加强对行政机关的监督检查。抓住损害群众切身利益的突出问题，切实纠正部门和行业不正之风。严肃查处违法违纪行为，严肃惩处腐败分子。

各位代表！站在新起点，实现新发展，是时代的呼唤，人民的重托，是历史赋予我们的光荣而神圣的使命。让我们紧密团结在以胡锦涛同志为总书记的党中央周围，高举邓小平理论和“三个代表”重要思想伟大旗帜，全面落实科学发展观，在省委、省政府和市委的坚强领导下，解放思想，干事创业，开拓创新，加快发展，为实现聊城跨越发展和率先崛起，为完成“十一五”规划的宏伟目标而努力奋斗！
